# Determination of Pentachlorophenol in Seafood Samples from Zhejiang Province Using Pass-Through SPE-UPLC-MS/MS: Occurrence and Human Dietary Exposure Risk

**DOI:** 10.3390/molecules28176394

**Published:** 2023-09-01

**Authors:** Xiaoyang Yan, Qiaoling Zhao, Zhongyong Yan, Xuechang Chen, Pengfei He, Shiyan Li, Yi Fang

**Affiliations:** 1 School of Food and Pharmacy, Zhejiang Ocean University, Zhoushan 316022, China; 2 Key Laboratory of Sustainable Utilization of Technology Research for Fisheries Resources of Zhejiang Province, Zhejiang Marine Fisheries Research Institute, Zhoushan 316021, China; 3 Zhoushan Institute for Food and Drug Control, Zhoushan 316012, China; 4 Zhejiang Marine Ecology and Environment Monitoring Center, Zhoushan 316021, China; 5 Zhejiang Fisheries Technology Extension Center, Hangzhou 310023, China

**Keywords:** pentachlorophenol, seafood, liquid chromatography-mass spectrometry, health risk

## Abstract

Pentachlorophenol (PCP) has attracted wide attention due to its high toxicity, persistence, and bioaccumulation. In this study, a sensitive UPLC-MS/MS method for the determination of PCP in seafood samples was developed and validated. The samples were ultrasonic extracted with acetonitrile containing 1% acetic acid-acetonitrile and followed by using a pass-through solid-phase extraction (SPE) cleanup on Captiva EMR-Lipid cartridges. The linearity of this method ranged from 1 to 1000 μg/L, with regression coefficients of >0.99. The detection limit and quantitation limit were 0.5 μg/kg and 1.0 μg/kg, respectively. The recoveries in different types of seafood samples ranged from 86.4% to 102.5%, and the intra-day and inter-day relative standard deviations (RSDs) were 3.7% to 11.2% and 2.9% to 12.1%, respectively (n = 6). Finally, the method has been successfully utilized for the screening of PCP in 760 seafood samples from Zhejiang Province. PCP was detected in 5.8% of all seafood samples, with the largest portion of detections found in shellfish, accounting for approximately 60% of the total. The average concentrations detected ranged from 1.08 to 21.49 μg/kg. The non-carcinogenic risk indices for adults and children who consume PCP ranged from 10^−4^ to 10^−3^ magnitudes. All of these indices stayed significantly below 1, implying that the health risk from PCP in marine organisms to humans is minimal.

## 1. Introduction

In recent decades, Pentachlorophenol (PCP) and its soluble PCP sodium salt (PCP-Na) have been produced commercially as active ingredients in herbicides, silicides, wood preservatives, fungicides, and bactericides. Their release into the environment has toxic effects on ecosystems [1,2]. China uses PCP and its sodium salt to eradicate parasitic schistosomes in paddy fields and ponds, where they are used in large quantities. Due to their high hydrophobicity, environmental persistence, and long half-life in water, they can accumulate in organisms through the food chain and are not easily biodegradable [3,4,5]. When PCP-Na enters an organism, it can convert to PCP. PCP has complex physiological toxicity, including carcinogenicity, endocrine toxicity, and genotoxicity [6,7]. PCP was classified as a carcinogen in the 2017 list of carcinogens published by the International Agency for Research on Cancer (IARC) of the World Health Organization [8]. On 23 February 2021, the European Union issued Regulation (EU) 2021/2772, which specifies the limits for PCP and its salts and esters in Annex I of the POPs Regulation [9]. In the United States, PCP is included in the Toxic Substances Control Act (TSCA), which restricts its production, import, processing, and use [10]. In Japan, PCP is listed under the Act on the Control of Toxic Substances (CSCL), and a license application is required for its production, import, use, and sale. According to Article 3 of the CSCL, PCP is classified as a specific toxic substance, and its scope and conditions of use are strictly limited. Additionally, PCP is also listed in the appendix of the Water Pollution Control Act, which prohibits its concentration in water bodies from exceeding 0.003 mg/L when discharged [11]. Announcement No. 235 was released in 2002 by the Ministry of Agriculture and Rural Affairs of the People’s Republic of China, explicitly prohibiting the detection of PCP in any food animals [12].

PCP is imported into the ocean through wastewater and rivers and is now also being detected to varying degrees in marine plants and animals such as algae, annelids, mollusks, and crustaceans, as well as in sediments [13,14,15]. In recent years, monitoring the levels of PCP in marine products has become an important aspect of aquatic safety. Therefore, it is necessary to test marine products for the presence of PCP.

At present, gas chromatography (GC) [16], liquid chromatography (LC) [17,18], gas chromatography-mass spectrometry (GC-MS) [19,20,21], high-performance liquid chromatography-mass spectrometry (HPLC-MS/MS) [22], and other methods are commonly used for detecting PCP and its sodium salt. However, GC and GC-MS require sample derivatization, which is somewhat cumbersome, and LC is not very sensitive. On the other hand, ultra-performance liquid chromatography-tandem mass spectrometry (UPLC-MS/MS) is widely used due to its selectivity, sensitivity, and simple pretreatment [23,24]. Liquid–liquid extraction [25] and solid-phase extraction [26] are the main means of sample pretreatment for PCP in animal-derived foods. However, these methods involve complex operating procedures and require excessive amounts of organic reagents. Additionally, the presence of various interfering substances, such as proteins and fats in fish, significantly affects the accuracy of the test results. In this study, the Captiva EMR-Lipid columns (EMR) are used to purify samples. The EMR column, alternatively referred to as the enhanced lipid removal purification column, utilizes a combination of size exclusion and hydrophobic interactions. This enables the selective and efficient elimination of lipids, particularly those with a carbon chain of C5 or higher. Furthermore, it significantly minimizes analyte loss, resulting in an improved method with enhanced reliability and durability. This technology eliminates the necessity for activation, elution, and other processes, thereby simplifying the sample pretreatment procedure. Additionally, it facilitates gravity flow and user-friendliness.

This study aims to establish a method for detecting the presence of PCP in various marine product samples. The method will be suitable for the mass screening detection of water products. To achieve this objective, the chromatographic conditions, extraction conditions, purification conditions, and others were optimized using the EMR column. By implementing this method, the PCP content in a wide range of seafood samples from the coastal areas of Zhejiang Province was determined, and their distribution characteristics were analyzed. Furthermore, a study on dietary health risk assessment was conducted.

## 2. Results

### 2.1. Optimization of the UPLC-MS/MS Analytical Method

#### 2.1.1. Determination of the Mobile Phase

PCP is an acidic and stable phenolic compound that has been halogenated. When compared to the acetonitrile mobile phase system, the PCP compound has a later peak time and a higher mass spectral response in the methanol mobile phase system. In order to improve the peak shape of the target compound, prevent peak drifting, and stabilize the peak time, ammonium acetate, a buffer salt, is added to the aqueous phase. The electrospray ionization source (ESI) in this mobile phase containing ammonium acetate demonstrates excellent sensitivity. Therefore, it was determined that the organic phase would be methanol, while the aqueous phase would be 5 mmol/L ammonium acetate.

#### 2.1.2. Selection of Chromatographic Columns

PCP is highly hydrophobic, and several studies have shown that C18 columns are commonly used as analytical columns [27,28]. However, variations in silica purity, bonding, and hybridization techniques between different brands of C18 columns can impact the sensitivity of mass spectrometry analysis, resulting in differences in retention performance and selectivity. This study determined that the retention ability of PCP differed among the various brands of C18 columns tested. The later the PCP peak time, the stronger the mass spectral response. Under the conditions used in our laboratory, the Waters Acquity UPLC BEH C18 Column showed the strongest retention capacity and highest mass spectral response when analyzing PCP. Therefore, we ultimately selected it as the chromatographic analysis column.

#### 2.1.3. Determination of Mass Spectrometry Conditions

**Figure 1:**
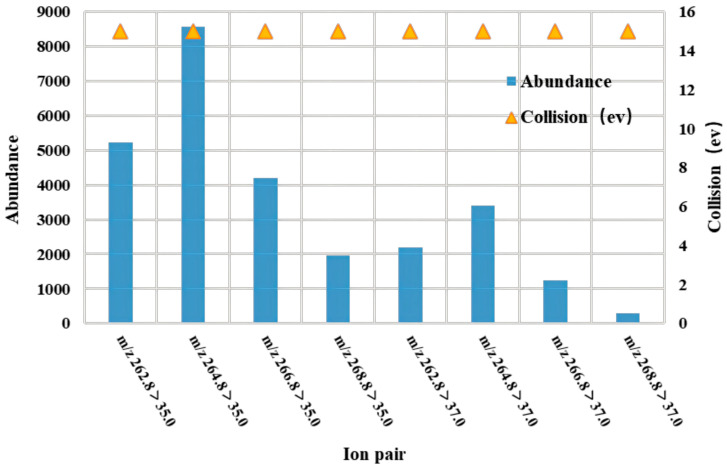
Comparison of response intensities for the 8 PCP ions.

**Figure 2:**
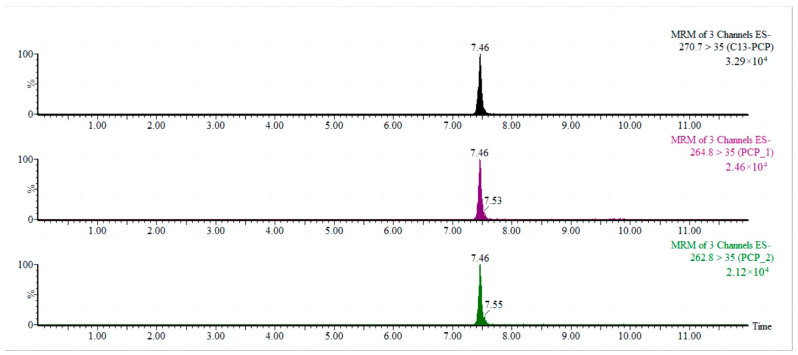
PCP MRM plots under optimized conditions.

The needle pump injection technique was used to perform a full scan of the primary mass spectra of ^13^C_6_-PCP and PCP-Na. Both ^13^C_6_-PCP and PCP-Na produced a strong response under negative source conditions and formed isolation clusters with four isotopic peaks (*m*/*z* 262.8, *m*/*z* 264.8, *m*/*z* 266.8, and *m*/*z* 268.8) in an approximate abundance ratio of 2:5:2:1, respectively. Further fragmentation of ions via secondary mass spectrometry revealed that they were [Cl]^−^ and had two isotopic peaks at *m*/*z* 35.0 and *m*/*z* 37.0, except for the ion *m*/*z* 262.8. The ion pair [M − Na]^−^ > [Cl]^−^ was therefore chosen as the optimized ion pair for the multiple reaction monitoring (MRM) mode. The collision energies for all eight pairs were optimized, and it was found that the optimal collision energy condition for all eight pairs was 15 eV. As shown in Figure 1, the *m*/*z* 264.8 > 35.0 ion pair had the highest abundance, followed by the *m*/*z* 262.8 > 35.0 ion pair. Four identification points were identified for the situation of two distinct parent ions corresponding to daughter ions, satisfying the qualitative requirements of EU Resolution 2006/657/EC [29] for the LC-^13^C_6_-PCP MS/MS analytical method. Figure 2 shows the PCP MRM.

### 2.2. Optimization of Pretreatment Conditions

#### 2.2.1. The Comparison and Optimization of Solid-Phase Extraction Columns

**Figure 3:**
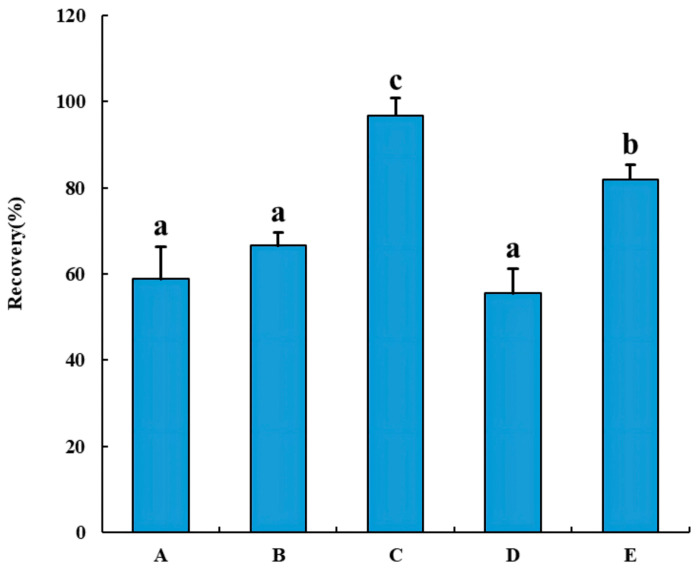
Effect of different purification methods on PCP recovery (A: Florisil SPE Cartridge; B: Si/PSA SPE GLASS Cartridge; C: EMR; D: PSA bulk; E: OasisMAX SPE). Data with different lowercase letters (a, b, c) in the graphs are significantly different (*p* < 0.05). Data are presented as means ± standard deviation (n = 3).

Seafood is known to contain high levels of protein, fat, and other long-chain substances that may potentially affect experimental results and shorten instrument life when co-extracted [30]. To investigate the recovery of sodium PCP, this study utilized four types of solid-phase extraction columns (SPE) for a comparison of recoveries: EMR, Florisil SPE, Si/PSA SPE, and OasisMAX SPE. Additionally, PSA dispersion solid-phase extractants were used. After analyzing the experimental results (Figure 3), it was found that the EMR recovery of 96.8% PCP in the four test columns was significantly different from the other four groups (*p* < 0.05). Following this, the OasisMAX SPE column showed a recovery rate of 81.9%. The results indicate that the EMR effectively removes fat and impurities from aquatic samples, and the extraction column also features fast filtration without activation during the experiment. Yang et al. [31] used the EMR extraction column for the first time in the detection of 17 fungicides in milk and dairy products, which not only reduced the experimental time but also improved the purification efficiency. EMR is used for purification purposes by adsorbing long-chain substances such as lipids and has been successfully used in various applications, such as detecting drug residues [27,30].

#### 2.2.2. Selection of Extraction Solvent and Extraction Volume

**Figure 4:**
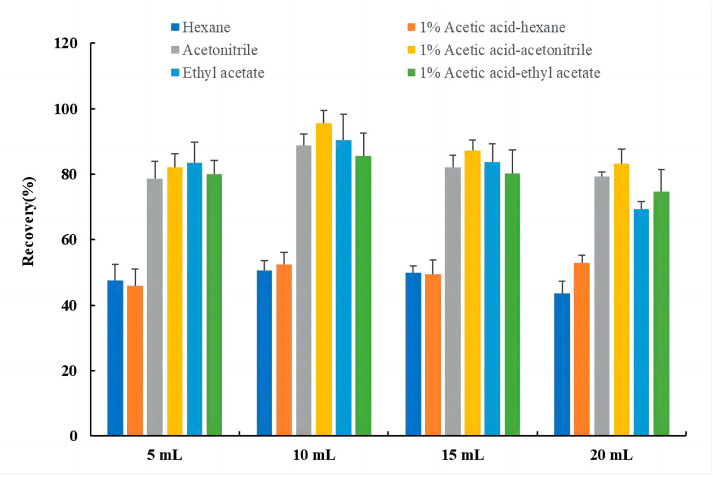
Validation results for different volume recoveries of 6 extracts. Data are presented as means ± standard deviation (n = 3).

In this experiment, six organic solvents were used for extraction, and each sample was extracted in triplicate under identical conditions. The extraction efficiencies of hexane, acetonitrile, ethyl acetate, 1% acetic acid-hexane (99:1, *v*/*v*), 1% acetic acid-acetonitrile (99:1, *v*/*v*), and 1% acetic acid-ethyl acetate (99:1, *v*/*v*) were evaluated. As the biological sample contained some water, 2 g of anhydrous magnesium sulfate was added to absorb the water and increase the extraction efficiency of the extractant. Ultrasound-assisted acid digestion was performed to further enhance the extraction efficiency, as the PCP in the sample was partially bound. The results in Figure 4 indicate that extraction using hexane, 1% acetic acid-hexane, ethyl acetate, and 1% acetic acid-ethyl acetate was unsatisfactory. Conversely, the recoveries of acetonitrile ranged from 78.6% to 88.8%, and the recoveries of 1% acetic acid-acetonitrile ranged from 82.1% to 95.5%, which were significantly higher than the other four extractants. Acetonitrile was found to have a positive effect on protein precipitation, and the addition of 1% glacial acetic acid made the acidified acetonitrile extract clearer and cleaner. Different volumes of 1% acetic acid-acetonitrile (5 mL, 10 mL, 15 mL, and 20 mL) were used, and the recoveries were 82.1%, 95.5%, 87.1%, and 83.2%, respectively. These results showed that the extraction using 10 mL of 1% acetic acid-acetonitrile was the most effective. Therefore, 1% acetic acid-acetonitrile and 10 mL were determined as the best extractant and optimum extraction volume, respectively, for this study.

### 2.3. Validation of the Method

The complex array of aquatic organisms found in the ocean means that matrix effects play a crucial role in UPLC-MS/MS analytical methods. Particularly in ESI mass spectrometry, the detection signal can be either hindered or boosted, which can compromise the accuracy and reliability of the analytical method [32]. This can negatively impact the sensitivity, precision, and authenticity of the chromatography-tandem mass spectrometry. To address this issue, PCP standard solutions were prepared in the mass concentration range of 1 to 1000 μg/L, and the linearity was evaluated by using the isotopic internal standard ^13^C_6_-PCP. The results showed that all PCPs had correlation coefficients ranging from 0.995 to 0.999, indicating good linearity in this concentration range and fulfilling the detection requirements of the instrument.

**Figure 5:**
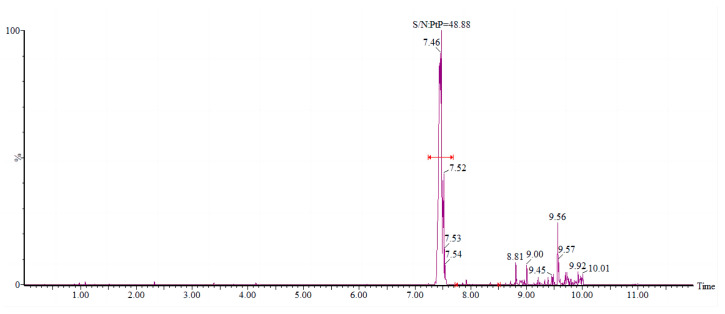
PCP 48.88× signal-to-noise ratio (S/N).

The spiked recoveries of fish, shellfish, shrimp, and crab samples were tested at levels of 1 μg/kg, 5 μg/kg, and 10 μg/kg. Each spiked level was repeated six times, as shown in Table 1. The levels of PCP found in 11 coastal organisms were spiked, ranging from 86.4% to 102.5%. Intra-day relative standard deviations of 3.7% to 11.2% and inter-day relative standard deviations of 2.9% to 12.1% were observed, confirming the accuracy, precision, and repeatability of the proposed method. The evaluation and calculation of the matrix effect (ME) for different types of seafood showed MEs of −9% to 11%, −15% to 10%, −17% to 13%, and 6% to 8% for fish, shellfish, crab, and shrimp, respectively. It is generally accepted that signal enhancement or inhibition is acceptable if the ME is above −20% or below 20% and conversely has a strong substrate effect [33,34]. The present study showed essentially no substrate effects for different species of marine organisms based on the results. The detection limit of the method is 0.5 μg/kg, determined through a 3-fold signal-to-noise ratio (S/N) calculation. The quantification limit of this method is determined by quantifying the chromatographic signal of ion pairs. It is obtained by multiplying the signal-to-noise ratio (S/N) of the labeled sample, which is not less than 1 μg/kg, by 10, as depicted in Figure 5.

## 3. Sample Analysis and Health Risk Assessment

### 3.1. Sample Analysis

PCP was detected in 760 seafood samples collected from coastal cities in Zhejiang Province, and 11 species of seafood were detected to varying degrees, indicating that the main source of PCP contamination in coastal Zhejiang Province is the large-scale illegal use of PCP in coastal mudflats and ponds during seawater fish farming for the purpose of killing snails and other marine organisms to clear the ponds. PCP in the surface soil of mudflats and ponds is easily combined with minerals and organic matter in the soil, thus fixing PCP contaminants in the sediment for a long time [35,36]. When shellfish, crabs, and shrimps are grown in the sediment in mudflats and ponds, PCP is absorbed and transported into the sediment, thus affecting human health and safety.

**Figure 6:**
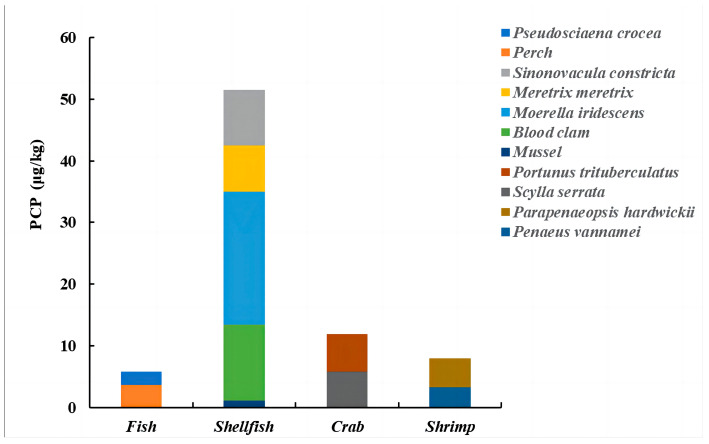
Mean concentrations of PCP detected in 11 seafood species.

The average concentrations detected in this study ranged from 1.08 to 21.49 μg/kg (Figure 6). The mean concentrations in *Pseudosciaena crocea* and *Perch* were 2.19 μg/kg and 3.61 μg/kg. The differences in concentrations in different species of fish may be related to their different habits and feeding habits. The mean concentrations for *Sinonovacula constricta*, *Meretrix meretrix*, *Moerella iridescens*, *Blood clam*, and *Mussels* were 9.01 μg/kg, 7.52 μg/kg, 21.49 μg/kg, 12.40 μg/kg, and 1.08 μg/kg, respectively, and it is worth noting that coastal mussels are cultured in net cages and floating cranes to avoid contact with sediment; therefore, the number of detections and concentrations was much lower than for other species. The mean concentrations for *Portunus trituberculatus*, *Scylla serrata*, *Parapenaeopsis hardwickii*, and *Penaeus vannamei* were 6.72 μg/kg, 5.86 μg/kg, 2.56 μg/kg, and 4.81 μg/kg, respectively. PCP was detected in 5.8% of the total in this study, with the highest proportion of detections in shellfish, accounting for about 60% of the total, and PCP was also detected in mud samples from the corresponding sea areas. In 2014, Feng [37] collected a total of 55 fish from the surrounding waters of Dongting Lake to conduct a phenol assay. These fish belonged to nine different species. The results showed that the mean concentrations of ∑PCPs in the samples ranged from 1.67 to 141.59 μg/kg, with a ∑PCP concentration of 234.65 μg/kg. Specifically, the mean concentrations of PCP in bighead carp, silver carp, and carp were found to be 8.07 μg/kg, 21.94 μg/kg, and 18.47 μg/kg, respectively. In comparison, the average concentration of PCP in the fish studied was lower, which suggests that it may be attributed to the open culture environment.

### 3.2. Health Risk Assessment

PCP is classified as a priority environmental organic pollutant and a persistent pollutant by the US Environmental Protection Agency (EPA). It evaluates both carcinogenic and non-carcinogenic risks associated with PCP. The US EPA has established acceptable risk levels for carcinogens ranging from 10^−6^ to 10^−4^. This means that the minimum acceptable carcinogenic risk level is 10^−6^, while the maximum is 10^−4^. In this study, the recommended risk assessment model for toxic and hazardous substances by the US EPA was used for quantitative assessment and was calculated as follows:

(1) $$EDI=\frac{C_{seafood}\cdot CR}{BW}$$

In the formula, *C_seafood_* is the measured level of PCP in seafood mg/kg. The estimated daily intake (*EDI*) of μg/(kg·d) represents the PCP consumption from seafood for residents. *CR* is the mean daily intake of seafood g/d. The resident body weight (*BW*) of adults and children in this study was 60 kg and 15 kg [38,39], and 142.2 g/d and 50 g/d of seafood, respectively [40,41].

The non-carcinogenic risk of PCP is expressed as a hazard quotient (*HQ*) and is calculated as follows:

(2) $$HQ=\frac{EDI}{RfD}$$

The carcinogenic risk of PCP is expressed as a carcinogenic risk value (*R*) and is calculated as follows:

(3) R=EDI×CSF

The *HQ* formula is a risk index that is non-carcinogenic and is utilized for identifying health risks that stem from exposure to toxic substances. When the *HQ* value exceeds 1, it signifies a potential health risk. Conversely, if the value is lower than 1, then exposure to PCP does not pose a significant health risk to the population. Conversely, *R* denotes the carcinogenic risk of PCP. If the value of *R* is less than 1 × 10^−6^, it implies that there is not any carcinogenic risk. Nonetheless, if the value of *R* exceeds this, then there is a certain degree of carcinogenic risk.

The carcinogenicity slope factor (*CSF*) for PCP is 1.2 × 10^−1^ [mg/(kg·d)]^−1^, and the reference dose (*RfD*) is 3 × 10^−2^ [mg/(kg·d)] [37].

**Figure 7:**
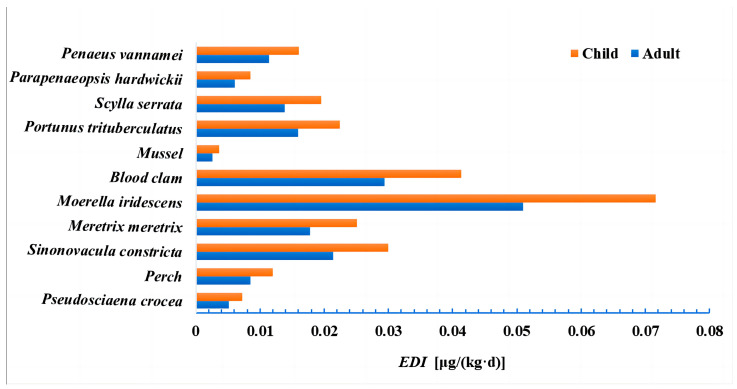
Resident daily exposure EDI [μg/(kg·d)].

Assuming that residents consume seafood on a daily basis, Figure 7 shows the *EDI* for residents. Among adults, shellfish had the highest *EDI*, followed by crab, shrimp, and fish. Among children, shellfish had the highest *EDI*, followed by crab, shrimp, and fish. All types of seafood showed significantly lower *EDIs* in adults compared to children, and the *EDI* of shellfish was significantly higher than that of fish, crab, and shrimp among the residents. In a toxicity study of razor clams, Qi et al. [42] noted that PCP-Na readily bioaccumulates in razor clams due to long-term exposure experiments. This finding indicates that shellfish are highly exposed to enrichment.

**Figure 8:**
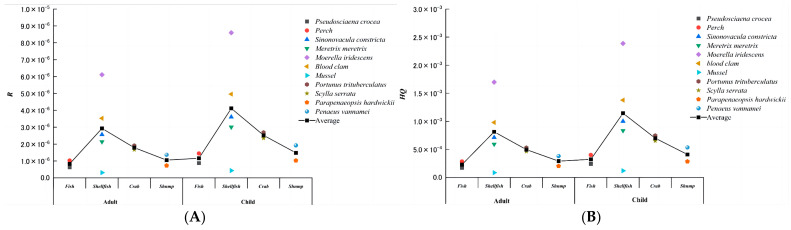
(**A**) PCP in different marine organisms is harmful to human carcinogenic health. (**B**) PCP in different marine organisms poses non-carcinogenic health hazards to humans.

PCP has some degree of carcinogenicity, calculated as follows in accordance with Section 3.2 (Figure 8A). The *R* for fish varies between *Pseudosciaena crocea* (6.2 × 10^−7^) and *Perch* (1.0 × 10^−6^), with an average of 8.2 × 10^−7^ for adults, and *Pseudosciaena crocea* (8.8 × 10^−7^) to *Perch* (1.4 × 10^−6^), with an average of 1.2 × 10^−6^ for children. For shellfish, the *R* ranges from *Mussels* (3.0 × 10^−7^) to *Moerella iridescens* (6.1 × 10^−6^), with an average of 2.93 × 10^−6^ for adults, and *Mussels* (4.3 × 10^−7^) to *Moerella iridescens* (8.6 × 10^−6^), with an average of 4.1 × 10^−6^ for children. The *R* for crabs ranges from *Scylla serrata* (1.7 × 10^−6^) to *Portunus trituberculatus* (1.9 × 10^−6^), with an average of 1.8 × 10^−6^ for adults, and *Scylla serrata* (2.3 × 10^−6^) to *Portunus trituberculatus* (2.7 × 10^−6^), with an average of 2.5 × 10^−6^ for children. For shrimps, the *R* ranges from *Parapenaeopsis hardwickii* (7.3 × 10^−7^) to *Penaeus vannamei* (1.4 × 10^−6^), with an average of 1.0 × 10^−6^ for adults, and *Penaeus vannamei* (1.0 × 10^−6^) to *Parapenaeopsis hardwickii* (1.9 × 10^−6^), with an average of 1.5 × 10^−6^ for children. The indices fall within the range of 10^−7^ to 10^−6^ data levels, with a slightly lower risk for adults than children. Although the *R* is generally believed to fall within the range of 1 × 10^−6^ to 1 × 10^−4^, which are globally established recommended values for acceptable risk, there is still a possibility of carcinogenic risk.

The non-carcinogenic risk was calculated, and the results are presented in Figure 8B. The non-carcinogenic risk indices for adults and children consuming PCP ranged from 10^−4^ to 10^−3^ orders of magnitude. These indices were all well below 1, indicating that the risk to human health from PCP in marine organisms is low. Looking at the non-carcinogenic risk from a single-species perspective, all samples were deemed acceptable. In other words, the risk posed by PCP on human health was not significant and was similar to the carcinogenic risk. Furthermore, the non-carcinogenic risk for adults was slightly lower than for children. Assuming the population consumes various types of seafood daily, the total non-carcinogenic risk for adults and children is 6.1 × 10^−3^ and 8.6 × 10^−3^, respectively, according to the formula, and the total non-carcinogenic risk is also acceptable. However, even with this assumption, the total non-carcinogenic risk remains low. In conclusion, PCP in marine organisms poses minimal risk to human health, and no action is required to limit public exposure. The total non-carcinogenic risk is acceptable, indicating that it does not significantly threaten human health.

## 4. Materials and Methods

### 4.1. Materials and Reagents

HPLC-grade methanol, acetonitrile, hexane, ethyl acetate, ammonium acetate, and acetic acid (≥97.0%) were purchased from Fisher Scientific. Anhydrous magnesium sulfate (analytical purity) was purchased from St. Louis, MO, USA. The ^13^C_6_-PCP standard (CAS: 85380-74-1) with a purity of ≥97% was obtained from Toronto Research Chemicals. The PCP standard solution (GBW(E)080475) was purchased from the China Academy of Metrology, and ultrapure water was obtained from a Milli-Q ultrapure water purifier. The EMR SPE (300 mg/3 mL, Agilent, Santa Clara, CA, USA), PSA bulk (100 g, per bottle), Florisil SPE Cartridge (1 g, 6 mL), and Si/PSA SPE GLASS Cartridge (500 mg/500 mg, 6 mL) were purchased from Shanghai Ampera Laboratory Technologies Ltd. (Shanghai, China).

### 4.2. Instruments and Equipment

The following instruments and equipment were used: Acquity^TM^ UPLC; Quattro Premier^TM^ XE Tandem Quadrupole Mass Spectrometer (Waters, Milford, MA, USA); Centrifuge 5810 high-speed centrifuge (Eppendorf, Hamburg, Germany); MS2 vortex mixer (IKA, Staufen, Germany); rotary evaporator (Ningbo Tianheng Instrument Factory, Ningbo, China); ultrasonic cleaner (Shenzhen Jiejun Cleaning Equipment Co., Shenzhen, China).

### 4.3. Preparation of Standard Solutions

Take the pentachlorophenol standard solution accurately, dissolve it, and dilute it with methanol to create a 1 mg/L standard stock solution. Store it in a dark place at a temperature of −18 °C for future use. Dilute the working solutions to 0.5, 1, 2, 5, 10, and 20 μg/L using different blank substrates. Test them under the conditions specified in 4.4. Create a standard working curve by plotting the sample mass concentration on the x-axis and the response value on the y-axis. Quantify it using an internal standard measurement strategy.

### 4.4. Sample Collection and Preparation

**Figure 9:**
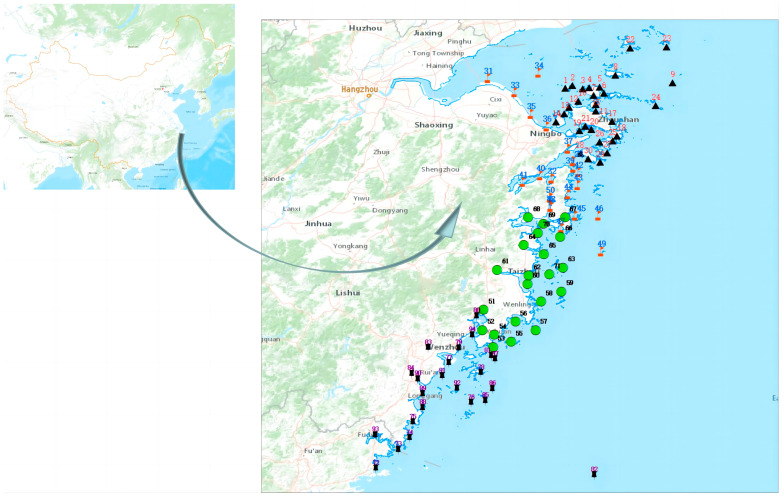
Distribution map for sample collection.

In March, May, and June 2022, a total of 760 samples were collected from the coastal regions of Zhoushan, Ningbo, Taizhou, and Wenzhou in Zhejiang Province. The collected samples were distributed to station sites located along the coastal areas of Zhejiang Province. Shellfish samples were collected from the tidal flats, whereas fish, shrimp, and crab samples were collected from marine aquaculture farms. The location for sample collection is shown in Figure 9. The symbols displayed in the Figure 9 represent various administrative regions. For instance, Zhoushan is represented by black triangles, Ningbo by red flags, Taizhou by green circles, and Wenzhou by black pushpins. The numbers within the Figure 9 correspond to location codes assigned for sample collection. Among these samples, 152 were fish (*Pseudosciaena crocea* and *Perch*), 400 were shellfish (*Sinonovacula constricta*, *Meretrix meretrix*, *Moerella iridescens*, *Blood clam*, and *Mussels*), 120 were crabs (*Portunus trituberculatus* and *Scylla serrata*), and 88 were shrimps (*Parapenaeopsis hardwickii* and *Penaeus vannamei*).

To prepare the samples for analysis, the fish were first scaled, and their skin was removed. The edible muscular part near the backbone was carefully extracted. For the shrimps, the heads were removed, the shells were discarded, and the intestinal glands were removed to obtain the edible portions. The shellfish and crabs were also de-shelled, and the muscle tissues were taken. The samples were cut into small pieces, homogenized in a tissue masher, and stored frozen at −18 °C.

To extract the samples, 2.0 ± 0.02 g of the homogenized sample was weighed and transferred to a 50 mL polypropylene centrifuge tube with a screw cap. Next, 100 μL of 100 μg/L of the ^13^C_6_-PCP isotope internal standard working solution was added to the sample. Afterward, 2.0 g of anhydrous magnesium sulfate and 10 mL of a 1% acetic acid-acetonitrile were added. The sample was then vortexed for 5 min and subjected to ultrasonic extraction for 10 min. After centrifugation at 8000 rpm for 5 min, the supernatant was extracted, and the residue was repeated once with 10 mL of 1% acetic acid-acetonitrile (*v*/*v*). The extracts were then combined in a rotary evaporation flask and evaporated at 38 °C until moistened. Next, 2 mL of 75% acetonitrile in water (*v*/*v*) was added, sonicated in a water bath, and left for purification, after which the entire solution was removed and passed through an EMR column (without activation). The filtrate was filtered by natural gravity and collected and then filtered through a 0.22 μm organic membrane for UPLC-MS/MS analysis.

### 4.5. Operating Conditions of the Instrument

The detection of PCP was carried out using a UPLC-MS/MS system that was equipped with an ESI. Separation was achieved using a Waters Acquity UPLC BEH C18 column (100 mm × 2.1 mm, 1.7 μm). The instrument chamber was maintained at approximately 20 °C, while the column was kept at 40 °C. Mobile phase A was a 5 mmol/L ammonium acetate solution, and B was methanol. The gradient elution procedure consisted of the following steps: starting with 40% B, an increase from 40% to 100% B was observed from 0 to 6.5 min, followed by maintenance of 100% B from 6.5 to 9.0 min, continued maintenance of 100% B from 9.0 to 9.5 min, a decrease from 100% B to 40% B from 9.5 to 12 min, followed by the use of 40% B till the end. An injection volume of 10 μL was used, and the flow rate was set at 0.2 mL/min.

The mass spectrometer was optimized with an ESI source operating in multiple reaction monitoring modes using negative ionization conditions. The capillary voltage was set to 2.5 kV, and the ion source was set to 150 °C. The desolvent gas was maintained at 500 °C, while the cone pore gas was set to 200 L/h, and the desolvent gas rate was set to 1000 L/h.

### 4.6. Method Validation

The LOQ was utilized as the reference point for the calibration curve to evaluate the method’s linearity and ensure dependable recoveries and reproducibility. All parallel experiments meeting a signal-to-noise ratio (S/N) of not less than 10 were repeated six times, and data were presented as mean values. The ME was determined by calculating the percentage, which is [(slope of matrix-matched standard curve/slope of the pure solvent standard curve) − 1] × 100%. The absolute recovery percentage was calculated as follows: [(peak area of the drug to be measured after purification of spiked sample/peak area of the drug to be measured in pure reagent solution of the same concentration as the spiked sample)] × 100%. To verify the accuracy of the analytical method, 1, 5, and 10 μg/kg of blank fish, shellfish, crab, and shrimp samples were added to determine the recovery (n = 6). The intra-day and inter-day precisions were evaluated by measuring the relative standard deviation (RSD) of samples of fish, shellfish, crab, and shrimp that were spiked and collected on the same day and on three consecutive days, respectively, in six replicates.

## 5. Conclusions

The UPLC-MS/MS method was used to detect PCP in seafood. The extraction and cleanup conditions of the EMR column were optimized, and the isotope dilution internal standard method was used for quantification, resulting in a significant improvement in sample recovery. The screening of PCP in 760 seafood samples from Zhejiang Province has been effectively implemented using this method. PCP was found in 5.8% of the total seafood samples, with average concentrations ranging from 1.08 to 21.49 μg/kg. The health risk assessment showed that the PCP found in the seafood samples was within acceptable limits. Furthermore, the health risk assessment revealed that PCP was slightly less concerning for adults than children.

## Figures and Tables

**Table 1:** Matrix effects, correlation coefficients, recoveries, relative standard deviations, and limits of quantification (LOQs) for PCP in 11 coastal organisms (n = 6).

Sample	ME (%)	R	LOQs (μg /kg)	1 μg/kg			5 μg/kg			10 μg/kg		
				Recoveries (%)	RSD(%) Intra-Day (n = 6)	RSD(%) Ntro-Day (n = 6)	Recoveries (%)	RSD(%) Intra-Day (n = 6)	RSD(%) Ntro-Day (n = 6)	Recoveries (%)	RSD(%) Intra-Day (n = 6)	RSD(%) Ntro-Day (n = 6)
*Pseudosciaena crocea*	−9	0.9972	1	92.2	6.5	4.6	90.6	8.6	9.2	90.9	7.5	9.7
*Perch*	11	0.9985	1	89.5	7.3	8.4	87.4	10.7	7.1	86.4	6.3	4.5
*Sinonovacula constricta*	6	0.9997	1	88.7	8.4	6.7	91.8	9.6	8.8	87.6	8.6	5.7
*Meretrix meretrix*	−14	0.9989	1	91.9	4.5	5.1	89.3	9.4	9.5	92.0	10.2	8.4
*Moerella iridescens*	7	0.9983	1	102.5	5.4	5.3	92.1	8.7	5.7	90.2	10.0	11.4
*Blood clam*	−15	0.9964	1	89.1	3.7	4.0	90.7	11.2	12.1	91.4	8.5	6.9
*Mussel*	10	0.9984	1	88.0	8.7	7.5	90.2	7.3	6.1	91.5	6.4	3.1
*Portunus trituberculatus*	13	0.9982	1	92.8	9.3	11.3	89.7	8.4	7.3	89.3	5.7	3.3
*Scylla serrata*	−17	0.9970	1	88.4	7.8	7.7	89.0	6.5	3.8	92.7	6.5	6.6
*Parapenaeopsis hardwickii*	6	0.9983	1	93.5	6.7	5.9	91.9	5.4	6.5	92.9	5.0	2.9
*Penaeus vannamei*	8	0.9975	1	95.2	5.8	5.6	92.7	7.6	7.1	93.2	7.3	5.0

## Data Availability

All the data are included in the paper.

## Sample Availability

Samples of the compounds are available from the authors.
